# Low rate of lymphedema after extended pelvic lymphadenectomy followed by pelvic irradiation of node-positive prostate cancer

**DOI:** 10.1186/1748-717X-8-271

**Published:** 2013-11-19

**Authors:** Elisabeth Rasmusson, Adalsteinn Gunnlaugsson, René Blom, Thomas Björk-Eriksson, Per Nilsson, Göran Ahlgen, Charlotta Jönsson, Karin Johansson, Elisabeth Kjellén

**Affiliations:** 1 Department of Oncology, Skåne University Hospital, SE-205 02 Malmö, Sweden; 2 Department of Oncology, Sahlgrenska University Hospital, SE-413 45 Gothenburg, Sweden; 3 Department of Urology, Skåne University Hospital, SE-205 02 Malmö, Sweden

**Keywords:** Prostate cancer, Lymphadenectomy, Pelvic irradiation, Node-positive, Lymphedema

## Abstract

**Background:**

The aim of the present study was to evaluate the prevalence and severity of lower limb lymphedema after pelvic lymphadenectomy and radiotherapy to the pelvic lymph nodes in patients with prostate cancer.

**Methods:**

Twenty-six patients underwent combined treatment for high-risk node-positive prostate cancer at Skåne University Hospital between April 2008 and March 2011. The treatment consisted of extended pelvic lymphadenectomy followed by androgen deprivation therapy and radiotherapy. The pelvic lymphnodes, prostate and seminal vesicles were treated with external beam radiotherapy (EBRT) to an absorbed dose of 50 Gy followed by a brachytherapy (BT) boost of 2x10 Gy to the prostate only. Twenty-two patients accepted an invitation to a clinical examination with focus on lower limb swelling. The median time between the end of radiotherapy and examination was 2.2 years (range 1.2–4.1).

**Results:**

Six patients (27%) experienced grade 1 lymphedema and two patients (9%) grade 2 while none had grade 3 or 4 according to the CTC Common Toxicity Criteria scale 4.0. Three patients required treatment with compression stockings.

**Conclusion:**

Brachytherapy and pelvic EBRT have a low incidence of lymphedema (at median 2.2 y after treatment) in patients with high-risk node-positive prostate cancer that have undergone pelvic lymph node dissection.

## Background

Lower extremity lymphedema is a complication, which has not been thoroughly studied in prostate cancer. It typically occurs after extended node dissection with additional radiation therapy, and it has a large negative effect on quality of life. There is little information about the rate of lymphedema after treatment. In a few studies the rate is mentioned to be 0-10% after extended lymphadenectomy (ePLND) [1]. In RTOG studies with extended-field irradiation for carcinoma of the prostate about 5% genital and/or leg edema has been noted and the edema remains chronic in the majority of the patients [2]. Lymphedema following treatment for gynecological cancer has been studied in more detail. The prevalence has been reported to be about 10-20% after pelvic lymph node removal but with large variations between different cancer sites [3-6]. The onset time seems to be short with the majority (>80%) of lower limb lymphedema diagnosed within 12 months after surgery [6].

Patients with high-risk prostate cancer represent about 20-35% of the newly diagnosed patients [7]. Mortality is high if patients are treated with non-curative intent. According to a Swedish nationwide database study published in 2011 the mortality at eight years for patients with locally advanced prostate cancer, Gleason score 8, treated non-curatively was 52% [8]. The incidence of node-positive disease is uncertain but has been reported to be 4-6% [9]. Node-positive disease has traditionally been considered to be equivalent to systemic disease and is therefore often treated with sole hormonal therapy; however, the opinion has changed lately towards a more aggressive attitude.

During the last decade many studies have shown significant decrease in cancer specific mortality when treating high-risk patients with combined systemic and local treatment with curative intention. The local treatment appears especially important for T3 or node-positive disease [10-12]. However, patients with Gleason pattern five have a high risk of clinical failure and death even after combined treatment with radiation therapy and hormonal therapy [13].

The number of involved lymph nodes and lymph node density (LND) are of prognostic value with cut-off values of 2–3 and 20%, respectively [9,12].

External beam radiation therapy (EBRT) combined with high-dose-rate brachytherapy (HDR-BT) boost, is an aggressive local treatment used for high-risk patients with good results [14-19]. In some of the studies pelvic lymph nodes have been included in the EBRT with acceptable toxicity [20].

The aim of this retrospective study was to evaluate late toxicity, with focus on lymphedema, in patients with node-positive prostate cancer treated at our hospital with ePLND followed by ERBT to prostate/seminal vesicles and pelvic lymph nodes and HDR-BT boost to the prostate. The study was carried out at the Department of Oncology at Skåne University Hospital in Malmö and Lund, Sweden and approved by the Central Research Ethics committee in Sweden.

## Materials and methods

A total of 26 patients with high-risk node-positive prostate cancer underwent combined treatment with HDR-BT and pelvic irradiation with EBRT following ePLND at the Department of Oncology at Skåne University Hospital between April 2008 and March 2011. Patient characteristics are shown in Table 1.

**Table 1:** Patient baseline characteristics

**Variable**	**No of patients (n=26)**	**%**
**Age at diagnosis (years)**		
≥70	11	42
60-70	13	50
<60	2	8
**Gleason score**		
6-7	6	23
8	6	23
>8	14	54
**PSA (at diagnosis)**		
<10	5	19
10-20	8	31
20-50	12	46
>50	1	4
**Prostate volume (at diagnosis)**		
<50	16	62
≥50	6	23
**No. of pos lymph nodes**		
1	15	58
2	8	31
3	2	8
**Lymph node density (% pos nodes)**		
<10	16	62
10-20	3	12
≥20	4	15
**T stage**		
1-2	5	19
3-4	21	81
**N stage**		
1	25	96
X	1	4
**HT**		
Neo-adjuvant	26	100
Adjuvant	23	88

HT= hormone therapy.

No patient had received previous radiation therapy (RT). All patients were classified as high-risk patients according to clinical stage ≥ T2c or Gleason score ≥8 or PSA >20. Patients were all scanned for metastases with bone scan and/or PET scan and were considered free from metastatic disease.

### Lymph node dissection

Twenty-five of the patients had undergone ePLND and had 1–3 positive lymph nodes. The anatomic extent of the lymphadenectomy followed general guidelines [21] and included dissection on but not lateral to the external arteries up to the ureteric crossing, obturator fossa and along the internal iliac artery. In two patients only the obturator fossa was dissected. One patient was not dissected due to pulmonary embolism at the time of diagnosis.

### Hormonal therapy

All patients received neo-adjuvant hormonal therapy (HT). Except for one case all patients received a GnRH-analogue, one was treated with anti-androgen and two patients were treated periodically with anti-androgen and GnRH-analogue. Flare prophylaxis with anti-androgen was provided. Median time between prescription of HT and start of radiotherapy was 5 months (range 3–9 months when excluding one outlier where the time interval was 8 years). Twenty-three patients (88%) received adjuvant HT. In most cases HT was planned for 2–3 years.

### Radiotherapy

The clinical target volume (CTV) of the EBRT included the prostate, the seminal vesicles and the pelvic lymph nodes. The planning target volume (PTV) was created by expanding the CVT iso-tropically with 8–10 mm margin. CTV was defined according to RTOG recommendation in most cases (18/22). However, large variations in CTV segmentation were however found in the reminder of the patients, due to the lack of firm guidelines in the start-up of this treatment. EBRT was given with 3D-CRT (n = 8), IMRT (n = 12) or helical tomotherapy (n = 2) to a total dose of 50 Gy in 2 Gy daily fractions, 5 days/ week. HDR-BT was given to the prostate gland with 2 mm margin in two 10 Gy fractions at a two week interval. The HDR-BT was started at median 13 days (range 1–22) after the EBRT was completed. Due to logistic reasons four patients received their HDR-BT in the middle of the EBRT session. All patients completed the intended course of radiation.

Median time between diagnosis (of prostate cancer) and start of radiation therapy was 8 months (range 4–102). Median time between lymph node dissection and start of radiation therapy was 5 months (range 3–100). Two patients had lympocele on planning CT. Radiotherapy details are presented in Table 2.

**Table 2:** Radiotherapy structure volumes (n=22)

**Structure/Volume**	**Mean volume (cm**^**3**^**)**	**Range (cm**^**3**^**)**
**PTV T**	158	83-272
**(prostate/vesicles**^**a**^**)**		
**PTV N**	711	161-1361
**(pelvic nodes**^**b**^**)**		
**Treated volume**^**c**^	1349	520-2107
**Irradiated volume**^**d**^	5832	3630-10810

^a^Vesicles included in PTV T in 7 (32%) patients.

^b^Defined according to RTOG in 18 (82%) patients.

^c^The volume of the body treated to a dose ≥ 47.5 Gy (95% of the prescribed dose).

^d^The volume of the body treated to a dose ≥ 25 Gy (50% of the prescribed dose).

### Lymphedema examination

All 26 patients were invited to participate in an examination concerning late side effects with focus on lymphedema. Twenty-two (85%) patients accepted. Of the four patients who refused to attend, one had metastatic disease while the others referred to other reasons.

Written information about the examination was sent to the participants together with a standard quality of life questionnaire for follow-up of patients treated for prostate cancer. Written consent to participate in the study was received at the visit. At the examination, patients were interviewed about any symptoms and comorbidity and they were physically examined by an oncologist. Health factors such as body mass index (BMI), smoking habits and exercise habits were registered. The latter were registered and classified according to IPAQ-2005 (International Physical Activity Questionnaire). Patients were also asked to assess their concern for relapse and how satisfied they felt with their treatment so far. Their current PSA was collected from hospital records.

A physiotherapist, specialized in lymphedema, examined the presence of lower extremity lymphedema following a protocol including measurements of limb volume and local tissue water, palpation and questions about symptoms related to lymphedema. A geometric volume method was used to determine limb volume. Total limb volume was calculated as the sum of geometric segment using the frustum model [22]. Difference between the limbs was expressed in percentage (lymphedema relative volume, LRV). Local tissue water was evaluated with a device that transmits an ultra-high frequency electromagnetic wave of 300 MHz into an open-ended coaxial probe in contact with the skin. An electrical parameter, TDC, direct proportional to tissue water content, was calculated [23].

If a patient was found to have lymphedema requiring treatment he was offered a new appointment. All symptoms were classified according to Common Toxicity Criteria scale CTC 4.0 when applicable.

## Results

Median follow-up time was 2.2 years (range 1. 2–4.1). Half of the patients had on-going HT at the time of examination. Their disease was under good biochemical control, and none of them had any clinical signs of prostate cancer. Concerning comorbidity, cardiovascular disease was common. About 60% had a history with hypertension, ischemic heart disease, TIA, arrhythmia and /or heart failure. The majority of the patients were physically very active.

Details are given in Table 3.

**Table 3:** Patient characteristics at time of examination

**Variable**	**No. of patients (n=22)**	**%**
**Follow-up time (years)**		
- 1-2	9	41
- 2-3	8	36
- 3-4	2	9
- 4-5	3	14
**Adjuvant HT**		
- On-going	11	50
**PSA**		
- <0,1	14	64
- 0,1-1	8	37
**Smoking**		
- On-going	3	14
- Previous	9	41
- Non-smokers	10	45
**BMI**		
- <25	10	45
- 25-30	10	45
- >30	2	9
**Level of exercise**		
- High	7	32
- Moderate	10	45
- Low	5	23
**Comorbidity**		
- Cardio-vascular disease	13	59
- Diabetes mellitus	3	14
- IBD	1	5
- Chronic pain	3	14
- None (of above)	2	9

IBD= inflammatory bowed disease.

HT= hormone therapy.

### Lymphedema

Six patients (27%) experienced grade 1 lymphedema and two patients (9%) grade 2 while none had grade 3 or 4 according to the CTC Common Toxicity Criteria scale 4.0.

There was no correlation between the reported radiotherapy volumes presented in Table 2 and lymphedema.

The measurements of LRV between the two lower extremities showed that that 5/22 patients had a LRV of more than 5%, two of them had no signs of lymphedema, one had severe osteoarthritis in one knee and suffered from chronic intermittent swelling. Two of these five patients had a LRV of more than 10%; one was born with a difference in size of the limbs and had only mild leg swelling.

No edema was registered based upon TDC measurements (0/20).

More than 50% of the patients reported on different symptoms from the legs such as swelling and pain. Two were treated with compression stockings before the visit and one patient was diagnosed with lymphedema requiring treatment and was provided with compression stocknings at the time of examination. Details are presented in Table 4.

**Table 4:** Lymphedema assessments

**Variable**	**No. of patients (n=22)**	**%**	**Comment**
**LRV**			
<5%	17	77	1 compression stockings
5-10%	3	14	
<10%	2	9	1 malformation
**Palpable increase of consistency**	8	36	
**Pitting edema**	4	18	
**TDC**	0	0	
**Compression stockings**			
Before assessment	2	9	
Initiated	1	5	
**Reported symptom (from patients)**			
Tension	5	23	3 legs, 1 knee, 1 knee/leg
Heaviness	4	18	3 legs & thighs, 1 leg
Swelling	11	50	5 legs, 3 thighs, 1 knee, 3 feet, 1 pubic, 1 knee/toe
Pain	6	27	1 sole, 1 limb, 4 thighs
Tingling	1	5	leg
Numbness	2	9	Sole, knee/leg/feet
Cramp	4	18	2 limb, 1 foot, 1 leg
Warmth	1	5	Knee/leg/feet
Life limiting symptoms	7	32	2 much, 2 partly, 3 a little

LRV=lymphedema relative volume.

TDC=tissue dielectric constant.

### Other side effects

Urinary side effects were common but usually mild, i.e. CTC grades 1–2. The most common side effects were signs of obstruction and frequency symptoms, and mild incontinence was quite common.

Rectal side effects were less common and also usually mild, i.e. CTC grades 1–2. The most common symptoms were intermittent rectal haemorrhage, urgency defecation problems in the morning, proctitis and faecal incontinence. One patient had faecal incontinence CTC grade 3.

Over 70% of the patients had total erectile dysfunction (ED) and the remaining had milder symptoms.

One patient reported pelvic pain CTC grade 3. This was a patient with multiple fractures in the pubic bone. The same patient had also lymphedema CTC grade 2.

Fourteen patients (64%) reported only very mild side effects, maximum CTC grade 1 except for ED.

Side effects are summarized in Table 5.

**Table 5:** Reported side effects according to CTC 4.0 (n=22)

**Variable**	**CTC1-2**		**CTC3-4**		**Comment**
	**No**	**%**	**No**	**%**	
**Lymphedema**	8	36	0	0	2-3 patients only volume difference between limbs
**Urinary**					
-Urinary obstruction	16	73	0	0	
-Urinary pain	2	9	0	0	
-Urinary frequency	14	64	0	0	
-Urinary incontinence	8	36	0	0	
-Urinary retention	3	14	0	0	
-Hematuria	1	5	0	0	
**Rectal**					
-Rectal pain	1	5	0	0	
-Proctitis	4	18	0	0	
-Fecal incontinence	3	14	1	5	
-Diarrhea	2	9	0	0	
-Rectal hemorrhage	6	27	0	0	
-Urgency defecation in the morning	6	27			No CTC-grading
**Other**					
-Erectile dysfunction	5	23	16	72	One missing value
-Pelvic Pain	2	9	1	5	Multiple pelvic fractures

CTC 4.0=Common Toxicity Criteria scale no 4.0.

### Concern from the patients

Ninety per cent of the patients did not worry at all or only worried a little about recurrence of the disease. Ninety-five per cent of the patients were very or quite satisfied with their treatment so far.

## Discussion

Results from this single-institution study show acceptable late toxicity after combined treatment of HDR-BT and pelvic irradiation with EBRT in patients with high-risk node-positive prostate cancer previously treated with extended pelvic lymph node dissection.

Most patients had only mild and manageable late side effects. A few patients had more marked inconvenience associated with the treatment such as lymphedema in combination with pelvic fractures and disabling fecal incontinence. This level of severe side effects is comparable with those found in other studies [17,24]. Most patients were very active, some even professionally.

Fifty percent of the patients experienced swelling in the lower extremities, mostly intermittent and the majority of them were not diagnosed with lymphedema. The volume difference between the limbs is a major factor in the CTC grading system. At least two patients had other medical conditions that explained the volume difference (osteoarthritis, malformation) but they were still scored as lymphedema. The true prevalence of treatment-related lymphedema in this group is thus probably even lower than presented in the results section, i.e. approximately 20% grade 1 and 5% grade 2 with three patients requiring treatment (14%). Even with this disadvantage we chose to use CTC 4.0 instead of the doctor’s opinion or radiological methods as this grading system is recommended for evaluation of the side effects of radiotherapy [25,26].

The definition of target volumes for radiation most likely influences the risk for lymphedema. In this study we did not find a significant relationship between segmented pelvic nodal volumes or treated/irradiated volumes and lymphedema. Note, however, that in three cases the nodal target volume were delineated far more caudally than the RTOG recommendations and these patients were all diagnosed with lymphedema.

Lymphedema rate could also be affected by a high proportion of cardio-vascular comorbidity and a relatively high proportion of overweight. However, these are common conditions in the population as a whole so the situation for the study population might give a good estimation of what to expect from this treatment. It seems important to register this side effect and initiate early intervention since patients could have a long survival and lymphedema tends to be chronic. Patients at risk for lymphedema could be diagnosed through examination after treatment or perhaps, even through a questionnaire sent to the patients a few months after treatment.

The high percentage of erectile dysfunction (ED) found in this study reflects the situation for elderly men when treated with long-term hormonal therapy in combination with radiation therapy. We did not have any baseline ED data in our study. Salomon et al. reported a baseline ED of 21.5% in patients diagnosed with clinically localized prostate cancer prior to radical prostatectomy [27].

The patients were generally very content with their treatment, probably influenced by the fact that their disease was under control and that they knew they had received a treatment with curative intent. The somewhat long neo-adjuvant HT is explained by the lack of stringent criteria for the systemic and local treatment of this group of patients.

The median follow-up time was quite short but earlier studies on lymphedema after lymphadenectomy and radiotherapy in patients with gynecological cancer have shown that the majority of lymphedema occurs during the first year after treatment [6]. A minor increase of the prevalence might therefore be expected after a longer follow-up.

Patients with high-risk nodal-positive disease have a high mortality if left untreated locally [8]. With an acceptable late toxicity this local treatment definitively seems favorable for patients in good clinical condition.

In conclusion, this study shows that the frequency of late complications including lymphedema was low in patients with high-risk node-positive prostate cancer treated with extended lymphadenectomy and combined irradiation with HDR-BT and pelvic EBRT.

## Abbreviations

EBRT: External beam radiotherapy; CTC 4.0: Common toxicity criteria scale 4.0; BT: Brachytherapy; ePLND: Extended lymphadenectomy; LND: Lymph node density; RTOG: Radiation therapy oncology group; HDR: High dose rate; GnRH: Gonadotropin releasing hormone; RT: Radiation therapy; CTV: Clinical target volume; PTV: Planning target volume; HT: Hormonal therapy; BMI: Body mass index; LRV: Lymphedema relative volume; ED: Erectile dysfunction.

## Competing interest

The authors declare that they have no competing interests.

## Authors’ contributions

ER has been involved in the acquisition of data, the examinations of the patients, the analysis of data and the main writing of the manuscript. AD has been involved in the design of the study, the examinations of patients and revising of the manuscript. RB has been involved in the design, the examinations and revising of the manuscript. TBE has been involved in the design and revising of the manuscript. PN has been involved in the statistical analysis and revising of the manuscript. GA has been involved in revising the manuscript. CJ has been involved in the design and performed the measurements concerning lymphedema. KJ has been involved in the design of lymphedema measurements and revising the manuscript. EK has been involved in the design, the examinations, the analyses of data and revising the manuscript. All authors have approved of the final manuscript.

## Authors’ informations

ER is an Oncologist at SUS (Skåne University Hospital) and Ms. C. in Chemical Engineering. AD is a Consultant Oncologist at SUS and Ph. D. in Oncology. RB is a Consultant Oncologist at SUS and Ph. D. in Oncology. TBE is a consultant Oncologist at Sahlgrenska University Hospital and Associate Professor at University of Gothenburg. PN is a Medical Physicist at SUS and Associate Professor at Lund University. GA is a consultant Urologist at SUS and Ph. D. in Urology. CJ is physiotherapist at SUS. KJ is physiotherapist at SUS and Ph. D in Oncology. EK is a Consultant Oncologist at SUS and Associate Professor at Lund University.
